# Synovial Sarcoma: A Clinicopathological and Radiological Study of 12 Cases Seen Over 18 Years

**DOI:** 10.4021/wjon2009.12.1201

**Published:** 2010-02-01

**Authors:** Soumaya Ben Abdelkrim, Amel Trabelsi, Faten Hammedi, Mohamed Zaher Boudagga, Ahlem Bdioui, Wafa Jomaa, Moncef Mokni

**Affiliations:** a Department of Pathology, Farhat Hached Hospital, Sousse, Tunisia; b Department of Medical Oncology, Farhat Hached Hospital, Sousse, Tunisia

**Keywords:** Synovial sarcoma, Radiology, Histology, Prognosis, Treatment

## Abstract

**Background:**

Synovial sarcoma is a rare malignant soft tissue tumor characterized by a poor outcome. We report herein our experience concerning synovial sarcoma and review its diagnosis, histology, treatment and prognosis.

**Methods:**

This is a retrospective review, from 1990 to 2007, of cases of synovial sarcoma diagnosed at the Department of Pathology, Farhat Hached hospital, Sousse, Tunisia. The clinical, radiological and pathological features as well as treatment modalities and patient's outcome were recorded.

**Results:**

From 1990 to 2007, 12 cases of synovial sarcoma have been diagnosed in our department. Patients’ mean age at the time of diagnosis was 21 years. There was no sex predominance and the lower extremity was the most commonly involved. A painful tumefaction was the most common presenting symptom. The duration of symptoms ranged from 6 months to 6 years. Malignancy was suspected on radiological findings in only 2 cases. Ten patients underwent surgery, in association with adjuvant chemotherapy in 4 cases, one of whom underwent post-operative radiotherapy. Histological subtypes included monophasic synovial sarcoma in 8 cases, biphasic synovial sarcoma in 3 cases and poorly differentiated synovial sarcoma in one case. At the time of analysis, 6 patients were dead with an average follow-up of 18 months.

**Conclusions:**

Synovial sarcoma is a rare malignancy with a propensity for young adults and a poor prognosis. Its symptomatology is non-specific and it is characterized by histopathological diversity. Diagnosis can be suggested by radiology and definitive diagnosis is achieved after pathological analysis.

December 18, 2009

## Introduction

Synovial sarcoma (SS) is a rare malignant neoplasm of mesenchymal origin; nevertheless, it is one of the most common malignant tumors occurring in soft tissue [1, 2]. The aim of this study was to review our experience with SS and to analyze its clinical, radiological and histopathological features. A short review of diagnosis, histology, treatment and prognosis of SS follows.

## Patients and Methods

The authors analyzed retrospectively clinical, radiological, histopathological and therapeutic features as well as outcome in a series of 12 cases of SS diagnosed at the Department of Pathology of Farhat Hached Hospital, Sousse, Tunisia, over a period of 18 years (1990 - 2007). Histological diagnosis was obtained by combining both morphological features and the immunoprofile of the tumors. Histological typing and subtyping was performed on hematoxylin and eosin stained sections, using the 2002 WHO classification of tumors of soft tissue and bone [3]. Grading was performed following the French Federation of Cancer Centers (Federation Nationale des Centres de Lutte Contre le Cancer: FNCLCC) grading system [4] which is determined by the sum of 3 scores attributed to tumoral differentiation, mitotic count and percentage of necrosis. A differentiation score of 3 is automatically attributed to SS, regardless of the actual morphologic degree of differentiation. As a result, a given tumor could be either grade 2 or grade 3, depending on mitotic rate, the extent of necrosis, or both.

## Results

**Figure 1:**
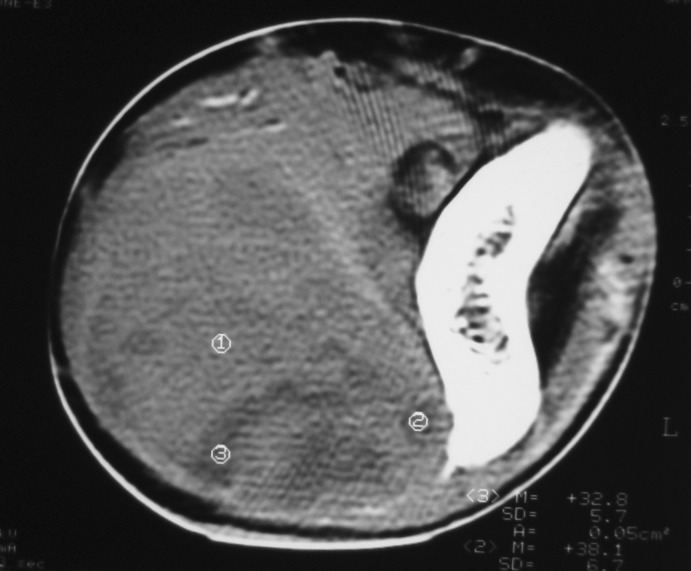
Unenhanced computed tomography image showing a solid soft tissue tumor with necrotic components.

**Figure 2:**
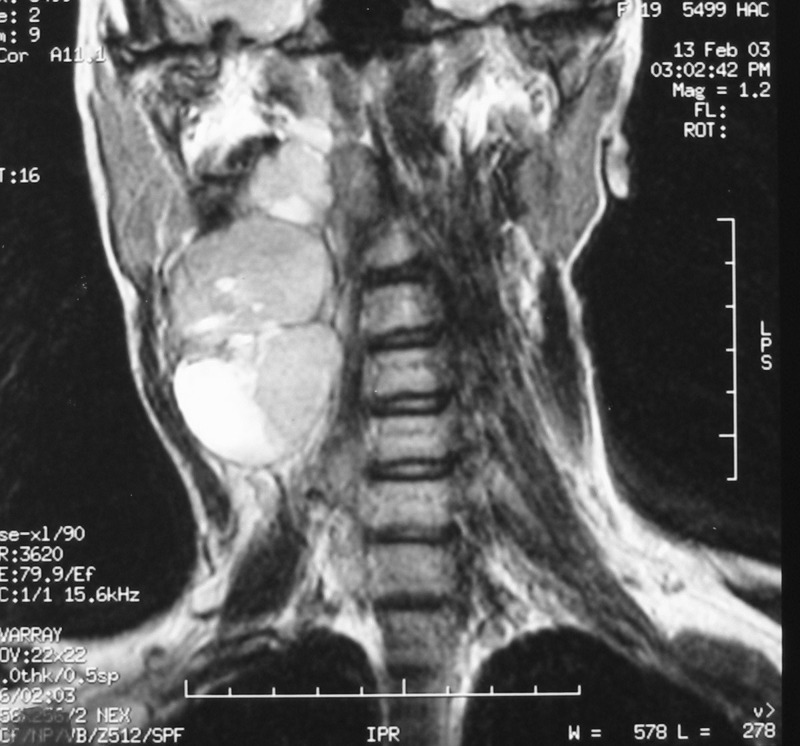
Coronal T2-weighted MR image showing a laterocevical tumor with heterogeneous signal.

There were 6 females and 6 males (sex ratio 1 : 1) ranging in age from 6 to 47 years (mean age: 21 years). Eight cases occurred in patients less than 20 years of age. The tumors occurred predominantly in the lower extremity (10 cases: foot in 4 cases, thigh in 4 cases and calf in 2 cases), one case arose in the upper extremity (elbow) and one case in the hypopharynx. The primary presenting symptoms comprised a painful tumefaction in 6 cases, isolated pain in 3 cases, dysphagia in the case of oropharyngeal SS and cough and expectorations in one case of SS revealed by lung metastasis. In the remaining case, SS was revealed by traumatism of the affected area. The mean time from onset of symptomatology and pathological diagnosis of sarcoma ranged from 2 months to 6 years. Clinical examination showed a painful tumefaction, firm in consistency, fixed to the deep plan, without cutaneous signs. Standard radiographs of the tumors were made in all the cases and showed calcifications in 2 cases, a well defined opacity in 3 cases, bone invasion in 4 cases and were interpreted as normal in 3 cases. Echography, performed in 6 cases, was assessed as normal in 6 cases and showed a heterogeneous mass in one case and a hypoechoic mass in one case. Computed tomography studies were undertaken at the first presentation in 7 cases and showed a heterogeneous mass with heterogeneous vascular enhancement (Fig. 1), invading adjacent bone in one case; calcifications were observed in 2 cases and the diagnosis of SS was suspected in one case. Magnetic Resonance Imaging (MRI) examination was performed primarily in 3 cases and showed a well defined mass enhancing asymmetrically after gadolinium injection (Fig. 2). The neoplasms were hypointense on T1-weighted MR images and hyperintense on T2-weighted MR images, vascular invasion was seen in one case and the diagnosis of SS was suspected in one case.

**Figure 3:**
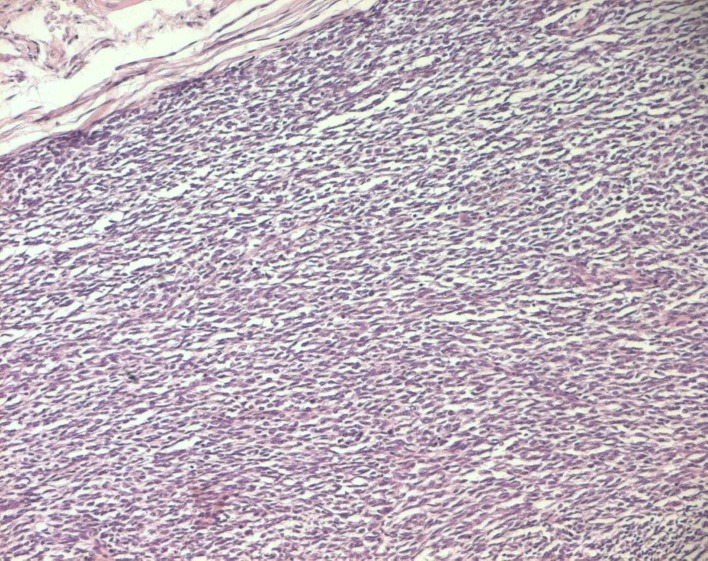
Monophasic fibrous SS: fascicles of spindle cells (HE x 100).

**Figure 4:**
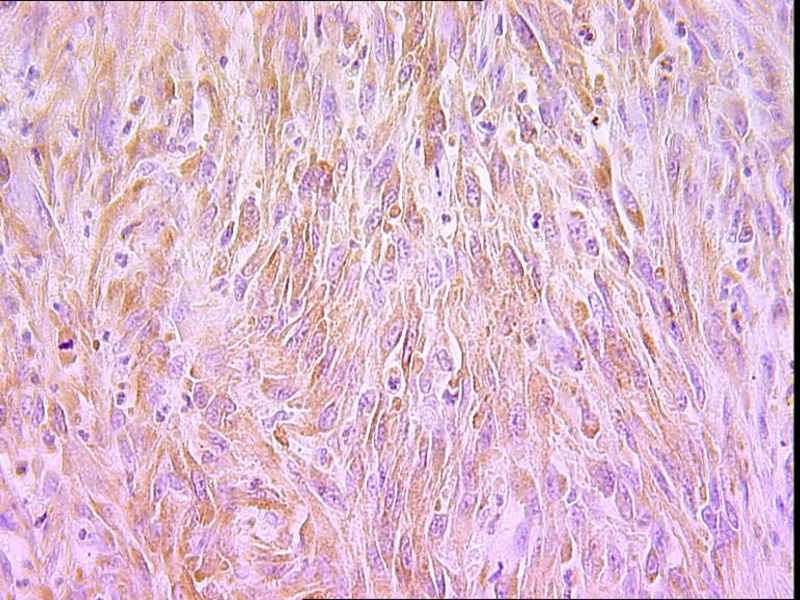
Spindle cells showing immunoreactivity for EMA (IHC x 400).

The histopathological study was performed on 8 surgical specimens and 4 biopsies. On gross appearance, tumors ranged in size from 3 to 17 cm (mean 8 cm), a diameter less than 5 cm was seen in only 3 cases. The tumors were soft or firm in consistency, grayish or whitish, well-circumscribed, encapsulated in 7 cases and showing cystic changes in 2 cases. Foci of necrosis were observed in 3 cases and macroscopic calcifications in 2 cases. On histological examination, calcifications were seen in 5 cases and foci of necrosis in 9 cases, osseous metaplasia was seen in one case. Histological subtypes included monophasic fibrous SS in 8 cases, biphasic SS in 3 cases and poorly differentiated SS in one case. There were no cases of monophasic epithelial SS. Monophasic fibrous SS showed a fascicular growth pattern with a variably collagenized stroma and a hemangiopericytoma-like vascular pattern (Fig. 3); biphasic SS showed, in addition to the abovementioned features, foci of glandular differentiation. Four tumors were grade 3 lesions and the 8 remaining cases were classified as grade 2 according to FNCLCC grading system. Immunohistochemically, the cases of biphasic SS were positive for Epithelial Membrane Antigen (EMA) and cytokeratin and negative for S100 protein and CD34. The cases of monophasic SS typically reacted at least focally with antibodies directed against EMA (Fig. 4), cytokeratin, vimentin and CD99. The poorly differentiated tumor had reactivity for S100 protein and vimentin and was negative for epithelial and muscular markers stains, it was otherwise morphologically and clinically compatible with the diagnosis of SS.

Surgical treatment was primarily applied in 9 cases, consisting in tumoral excision in 8 cases and amputation in one case. It was followed by chemotherapy in four cases and one of these four patients underwent post-operative radiation therapy. Different chemotherapy regimens were used due to changes in standard management over the years. The treatment was only chemotherapy in one case and one patient refused the treatment. Therapeutic abstention was applied in one case. Follow up information revealed that 3 patients were dead of advanced disease in an average period of 13 months (range 4 - 24 months). Local recurrence was seen in 6 cases, 3 of which developed documented metastases in an average period of 10 months (pulmonary, osseous, cutaneous and lymph nodes metastases). Two out of these 3 patients died one year and two years and a half respectively after the diagnosis. The 6 recurrent cases had all inadequate surgical margins. Three out of the 12 patients of our series were lost to follow up.

## Discussion

SS is a relatively rare malignancy, which accounts for approximately 5 - 10% of all soft tissue sarcomas [5]; nevertheless it is the fourth most common malignant tumor occurring in soft tissue after malignant fibrous histiocytoma, liposarcoma, and rhabdomyosarcoma [1]. The term SS stems from early literature as a result of the tumors microscopic resemblance to developing synovium [6]. Although many of SSs originate in close proximity to major articular structures, they uncommonly arise in joint cavities and it is no longer thought that these lesions originate from synovial tissue, but rather from a primitive mesenchymal cell [2]. SS is seen most commonly in the range of 15 - 40 years of age, but may occur at any age [6, 7]; males and females are almost equally affected [8]. It arises most frequently in the extremities [9], predominantly at the lower extremities [10]; minorities of the tumors have been reported to occur in other anatomical locations, with only 3% arising from the head and neck region [9]. A palpable mass is the most common presenting finding, but in some cases localized pain may precede the presence of a mass for many years [11]. Pain is often present and some have stated that SS is more likely to be associated with pain when compared to other soft-tissue malignancies [12]. SS have common radiological features with a variety of both benign and malignant lesions. However, there are some imaging findings that can suggest a pre-biopsy diagnosis of SS [13]. Radiologically, these masses may be ill-defined, lobulated or rounded; they are often very large and they usually present as a heterogeneous soft-tissue mass close to a joint, associated with calcification, usually peripheral, in as many as 30 - 50% of cases [7, 14]. Calcification may distinguish this lesion from other soft tissue sarcomas [15]. MRI signal characteristics are variable, the mass is isointense to muscle or hypointense on T1-weighted images, with or without some hyperintensity areas suggesting hemorrhage; on T2-weighted images, the tumor is said to show heterogeneous intensity because of necrosis and cystic degeneration. The tumor shows evidence of hemorrhage in 40% of the cases and infiltration of adjacent skeletal elements in 21 - 28% [16, 17]. Infiltrative margins, inhomogeneous signal intensity on T1- and T2-weighted images with solid portions, as well as septated areas of hemorrhage and necrosis represent a typical MRI appearance. Viable tumor tissue usually shows intense contrast enhancement [17]; however, especially in smaller tumors there is a reported tendency to present with well-defined margins and homogeneous signal on MRI images, possibly leading to the erroneous diagnosis of a benign lesion [18]. SSs are classified into 3 histologic subtypes, monophasic, biphasic and poorly differentiated. Histologically, the monophasic type contains oval to spindle-shaped cell population arranged into interlacing fascicles with tapering nuclei and a pale, scanty, poorly-defined cytoplasm [2]. A monophasic epithelioid variant of SS in which the epithelial component predominates has been described, but only a few cases of this type have been reported in the literature [19]. Biphasic SS shows 2 tightly linked histological patterns: a proliferation of fusiform cells of typical monophasic SS and well-differentiated glandular like formations. The poorly differentiated type resembles the small round monomorphic cells seen in Ewing’s/ primitive neuroectodermal tumour, but is identified as SS by its characteristic immunohistological, ultrastructural and cytogenetic features [6]. Poorly differentiated tumors are defined by FNCLCC criteria and include round cell morphology and high mitotic rate. Gland-forming biphasic and spindle cell fibrous monophasic tumors are the most common subtypes [20]. Monophasic spindle cell SS appears significantly more often than the biphasic form [19]. SS can show hyalinized stroma, mast cell influx, hemangiopericytoma-like vasculature, focal myxoid change, calcifications, cystic changes. Unusual histological features including Verocay body-like formations, vague rosettes, well-formed papillary structures, adenomatoid change, and rhabdoid morphology are sometimes observed [21]. Histologic and immunohistochemical findings are often sufficient to separate SS from other lesions such as malignant peripheral nerve sheath tumor, fibrosarcoma, and leiomyosarcoma [19]. Immunohistochemistry provides in fact a great contribution in the diagnosis of SS. Almost all biphasic SSs react for keratin markers or EMA in the epithelial component, whereas monophasic SSs have been reported to react in 60 - 70% of the cases. Immunoreactivity for S100 protein has been reported in 30% of the cases. The CD99 antigen has been reported to be expressed, however this is of limited clinical use because many spindle cell lesions react for the CD99 antigen [19]. Cytogenetically, SS is characterized by the reciprocal chromosomal translocation t(X;18)(p11.2;q11.2), involving fusion of the SYT gene located on chromosome 18 (18q11) to either the SSX1, the SSX2 or the SSX4 gene located on chromosome X, (Xp11) [22]. This translocation has been found in as many as 90% of SSs [23]. No other types of sarcoma have been found to carry the SYT-SSX fusion gene, then, analysis of the SYT-SSX fusion gene is now becoming a reliable tool for the pathological diagnosis of SS [22].

Treatment should be interdisciplinary. The standard treatment for localized disease is surgery; initial surgical management with adequate surgical margins by experienced surgeons for SS, preferably at specialized hospitals, should be considered to increase local control and improve outcome [24]. Radiotherapy has a well established role in improving local control but while surgery and radiation therapy have achieved excellent local control, distant metastasis remains the principal problem limiting survival [25]. SS is a chemosensitive soft tissue sarcoma [6] and adjuvant chemotherapy contributed to the improved long-term survival. Ifosfamide based chemotherapy has been associated with an improved survival in patients with SS [25]. Five to 10-year survival rates range from 25.2% to 76% and 11.2% to 38.2%, respectively [12, 26, 27]. The natural course of SS in characterized by a high frequency of local recurrences and/or metastatic disease. Metastatic disease occurs in 50% of patients [6]. There is a relatively high rate of late metastasis seen in patients with SS, in contrast to most other soft tissue sarcomas where metastases usually occur within 18 to 36 months of initial presentation [28]. As in other types of sarcoma, the most common site of metastasis is the lungs, occurring in 75% [14]. However, unlike most other types of sarcoma, synovial sarcoma also can metastasize to lymph nodes and bone marrow [2].

A variety of clinical and pathologic features are associated with an adverse outcome in patients with SS. These features include increasing age over 50 years; tumor size larger than 5 cm; extent of disease on initial presentation; local recurrence; the presence of poorly differentiated histology; grade 3 tumors; and microscopic positive surgical margins. Other prognostic factors include male sex; tumor necrosis; vascular invasion; rhabdoid cells; high mitotic rate; MIB1 index and DNA aneuploidy. Recent studies have claimed that adequate surgical margins and adjunctive chemotherapy and radiation therapy have improved prognosis [2, 5, 6, 8, 29-31].

In conclusion, SS is a relatively rare soft tissue malignancy of mesenchymal origin that arises most frequently in the extremities of young individuals. It is treated with surgery, when feasible, with adjuvant radiation therapy and chemotherapy. The improved survival with advanced disease may be attributable to its chemosensitivity and improved chemotherapy regimens.
